# Immersive Virtual Reality in Post-Stroke Rehabilitation: A Systematic Review

**DOI:** 10.3390/s23031712

**Published:** 2023-02-03

**Authors:** Andrea Demeco, Laura Zola, Antonio Frizziero, Chiara Martini, Arrigo Palumbo, Ruben Foresti, Giovanni Buccino, Cosimo Costantino

**Affiliations:** 1 Department of Medicine and Surgery, University of Parma, 43126 Parma, Italy; 2 Department of Diagnostic, Parma University Hospital, 43126 Parma, Italy; 3 Department of Medical and Surgical Sciences, University of Catanzaro “Magna Graecia”, 88100 Catanzaro, Italy; 4 Division of Neuroscience, IRCCS San Raffaele, University Vita-Salute San Raffaele, 20132 Milan, Italy

**Keywords:** virtual reality, stroke, rehabilitation

## Abstract

In recent years, next to conventional rehabilitation’s techniques, new technologies have been applied in stroke rehabilitation. In this context, fully immersive virtual reality (FIVR) has showed interesting results thanks to the level of immersion of the subject in the illusional world, with the feeling of being a real part of the virtual environment. This study aims to investigate the efficacy of FIVR in stroke rehabilitation. PubMed, Web of Science and Scopus were screened up to November 2022 to identify eligible randomized controlled trials (RCTs). Out of 4623, we included 12 RCTs involving post-acute and chronic stroke survivors, with a total of 350 patients (234 men and 115 women; mean age 58.36 years). High heterogeneity of the outcomes considered, the results showed that FIVR provides additional benefits, in comparison with standard rehabilitation. In particular, results showed an improvement in upper limb dexterity, gait performance and dynamic balance, influencing patient independence. Therefore, FIVR represents an adaptable, multi-faceted rehabilitation tool that can be considered in post-stroke rehabilitation, improving the compliance of the patients to the treatment and increasing the level of functioning and quality of life of stroke survivors.

## 1. Introduction

Stroke is one of the major causes of death and disabilities in the world and the reason for 116.4 million disability-adjusted life-years (DALYs) [1,2]. The Global Burden of Diseases, Injuries, and Risk Factors Study reports that, in 2016, stroke was responsible for 5.5 million deaths globally. The location of the brain damage, its extent and the amount of the recovery are key-points in the stroke’s final outcome [3]. Stroke survivors manifest deficits in physical functions, e.g., motor impairment in up to 80% of cases, associated with disability in language, sensory, behavioral and visual functions, dysphagia and cognitive domain (difficulty in intellectual capacity, memory, attention, orientations, awareness) [3,4].

Muscle functions and related movement are affected [4] and clinically perceived as modification of power and tone. The reduction of motor control can affect the trunk and upper and lower limbs and therefore influences the capacity of reaching, grasping and walking. This spectrum of deficits affects the activities of daily living (ADLs) and the patient’s self-sufficiency, with a great impact on patients’ quality of life [5,6].

In this context, stroke rehabilitation plays a key role, mainly if started early to counteract the effects of the disease. The primary goal is to improve quality of life through the prevention of physical function’s worsening, optimizing the residual capacities to improve performance and participation to social life. A successful rehabilitation program depends on the stroke’s features (such as severity, type, site) and the patient’s age, general condition and pre-stroke function [7].

The first three months after a stroke are the most critical for neurological recovery regarding upper and lower limb and “higher cerebral functions”; thus, an early, intensive and personalized rehabilitation plan is recommended [8]. Teasel et al. report that even after 6 months after stroke a patient can improve in functional outcomes [5].

Early recovery, even if incomplete, is largely due to the autonomous capacity of brain to recuperate, whereas in later phases the retrieval of lost function in case of brain damage is based on cortical reorganization and brain plasticity [5,9]. This is the focus of the neurophysiological approach in stroke rehabilitation based on mental practice and the cognitive rehearsal of physical movements that target the central representation of the movement to increase motor performance [10,11].

In recent years, next to conventional rehabilitation’s techniques, new technologies, such as virtual reality (VR), have been developed to enhance reorganization of the neuromotor ways and reduction of motor disability [12]. Virtual reality can be defined as “a medium composed of interactive computer simulations that sense the participant’s position and actions and replace or augment the feedback to one or more senses, giving the feeling of being mentally immersed or present in the simulation (a virtual world)” [13]. Thanks to the distinctive characteristics of the surroundings created by the system and the multiple sensor-based interactions between the subject and the simulator, a virtual scenario can be perceived as a realistic experience. In a virtual environment, the therapist can build, adjust and propose exercises that in conventional practice are unsafe, difficult to realize or too expensive. Moreover, due to the possibility of a gamification of the therapy, patients show more enthusiasm during a virtual experience in comparison to the tasks’ repetition of standard care rehabilitation, increasing patient’s compliance [14,15]. The use of multi-sensorial stimuli and challenging levels motivate the patients, which is one of the important elements to continue the treatment and improving rehabilitation outcomes [16]. Another key feature of virtual reality is the “sense of presence” [17]: a subject immersed in the illusional world reacts as if it was a real part of the artificial place and acts as if everything was real with a physiological, conscious and non-conscious emotional involvement [18], e.g., a virtual stressful experience is seen to increase the heartbeat [17]. Moreover, the degree of integration can be so intense that it could also influence pain reaction, with a pain alleviation during an immersive virtual reality distraction [18,19]. 

The immersion is a fundamental aspect of virtual reality and is correlated with the technology used by the system [20,21]. Features that characterize an immersive scenario involve the “graphics frame rate”, the “overall extent of tracking” (what body movements are detected), “tracking latency” (latency between head adjustment and relative modification in the simulation), the “quality of the images”, the “field of view” (dimension of the visual field of view versus normal vision and the extent of the displays encircling the user), the “visual quality of the rendered scene”, the “dynamics” and the “range of sensory modalities accommodated” [22]. In particular, tracking the user represents one of the most difficult challenges in rehabilitation [23]. Moreover, game design represents an essential aspect, improving the focus of the patient on the goal, keeping the patient engaged with indications and feedback [14].

Based on the level of immersion, there are three kinds of virtual reality: non-immersive, semi-immersive and fully immersive. A non-immersive virtual environment is commonly experienced in two-dimensions and is delivered through a computer display or console game system [23]. The subject can interact with the environment shown on a screen through tools, e.g., mouse, joysticks, Cybergloves/Cybergrasps or force sensors [24]. The perspective is allocentric (third person), and an avatar is displayed on the monitor [13].

A semi-immersive system is based on three-dimensional images created through “stereoscopic projections or displays with a fixed visual perspective” [25]. Users operate in the simulated environment with a deeper sense of connection and interactivity than in a non-immersive dimension due to sensors for subjects’ moves [26,27].

**Figure 1:**
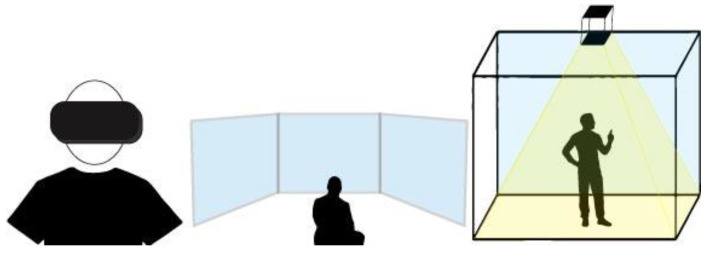
A representation of a head-mounted display, large screen projection system and cave automatic virtual environment.

In fully immersive virtual reality, subjects could operate egocentrally in a surrounding simulated world [28]. Various devices allow a real-time interaction between the visualized images and the head and body movements, reproducing the interactions of the real world [17] with a visual perspective depending on the head shifts [27]. Images can be presented through a head-mounted display (HMD), large screen projection system (SPS) or a cave automatic virtual environment (CAVE). The HMD is a wearable tool constituted by two little displays (located near the eyes, inside goggles or a helmet), a head-locating sensor (to adapt visualized images to head movements) and headphone for auditory signals. Recently, HMDs have also sensors that can detect hand movements so the communication with the virtual system can increase [17]. SPSs are large screens in which the virtual world is displayed [29]. The CAVE is a room where there are four or six monitors that, along with 3D glasses, offer an endless representation of the virtual worlds, integrated with a head-locating sensor and speakers for acoustic stimuli [17], see Figure 1.

Because of this continuous technologic development, studies using virtual environment to assess and treat medical conditions are increasing. Considering the last five years, several systematic reviews have been published about the application of the different kinds of virtual reality in neurological diseases: spinal cord injury [30], Parkinson’s disease [31,32,33,34,35,36,37,38], cerebral palsy [39,40,41], brain injury [42,43], stroke [44,45,46,47,48,49,50,51,52,53,54,55,56,57,58,59,60,61] and multiple sclerosis [62,63]. The aim of this study was to realize a systematic review to investigate the role of fully immersive virtual reality (FIVR) in stroke rehabilitation.

## 2. Materials and Methods

This systematic review was run and reported in conformity with the PRISMA statement (Preferred Reporting Items for Systematic Reviews and Meta-Analyses) [64].

### 2.1. Databases and Search Strategy

We explored three electronic databases: PubMed, Web of Science and Scopus. We selected studies published between 1 January 2010 and November 2022.

The purpose of this study was to analyze the effects of a fully immersive virtual reality training, alone or associated with mirror therapy or treadmill training or with conventional therapy, on motor impairment in comparison with standard care rehabilitation in patients with a stroke diagnosis.

We followed the PICO method (patients/population, intervention, comparison, outcomes): people with post-acute, sub-acute or chronic stroke (P); immersive virtual reality rehabilitation (I); comparison between FIVR rehabilitation and conventional rehabilitation, comparison between FIVR rehabilitation combined with conventional therapy and conventional rehabilitation alone (C); modification in motor function (in terms of upper extremity function, balance, gait and mobility ability) from pre- to the post-treatment and from pre-training to the follow-up evaluation (O).

Search terms included the following keywords connected with Boolean operators (AND/OR): virtual reality, immersive virtual reality, virtual environment, augmented reality, rehabilitation, intervention, treatment, therapy, training, stroke, cerebrovascular accident, hemipl*, hemip*. Moreover, a manual search of reference lists of selected papers was performed to identify additional relevant studies.

### 2.2. Selection Criteria

We included studies that fulfill the following criteria:

- Stroke patients (post-acute, subacute or chronic stroke);
- Randomized control trials (RCT);
- FIVR (head-mounted display or large screen projection or CAVE);
- Concerning motor impairment recovery;
- Written in English.

Exclusion criteria were:

- Observational, retrospective and cross-sectional studies, case reports, case series, case studies, reviews and meta-analysis;
- Studies involving only healthy subjects;
- Studies planned only for cognitive rehabilitation;
- Studies focused on cognitive disease (e.g., psychiatric disorder, dementia, mild cognitive impairment) or not regarding stroke (e.g., multiple sclerosis, Parkinson’s disease, spinal cord injury, traumatic brain injury, pain, cerebral palsy);
- Full-text not accessible through our institutional University Library System.

### 2.3. Data Extraction and Analysis

Two reviewers independently screened articles by title and abstract. Articles unclear from their title or abstract were reviewed according the selection criteria through full-text. Two reviewers extracted the data from selected studies using a standard form. The following information was extracted for each article: author and year of publication; characteristics of the participants (age, sample size, time after stroke); description of the intervention in the experimental group (virtual reality); description of the intervention in the control group; outcome measures; follow-up.

The data regarding outcomes, participants and intervention were not adequately uniform; therefore, it was not possible to draw up a meta-analysis. 

### 2.4. Assessment of Risk of Bias

The level of evidence of included studies was stratified according to the Oxford Center for Evidence-Based Medicine (OCEBM) [65]. Two authors independently assessed the methodological quality of data acquisition using the Critical Appraisal Skills Program for Diagnostic Test Studies (CASP) [66]. In case of disagreement, a third opinion was sought.

## 3. Results

### 3.1. Evidence Synthesis

#### Overview of the Trial Flow

**Figure 2:**
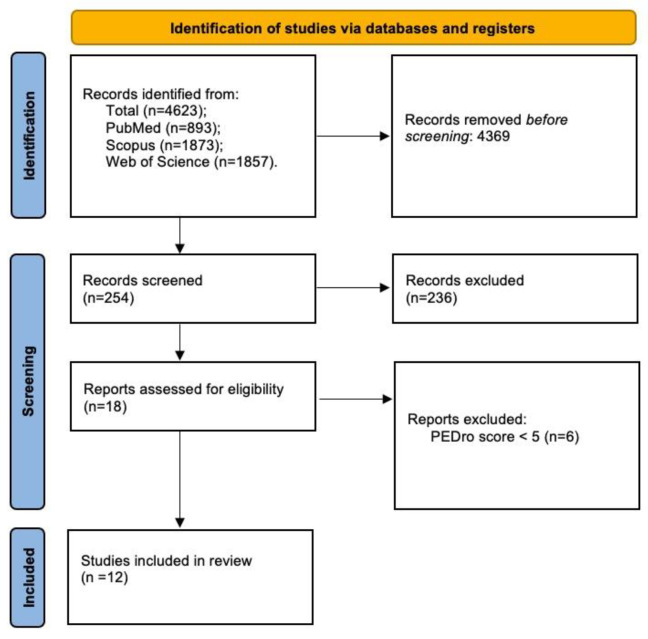
PRISMA flow diagram for Systematic Review.

The search on Pubmed generated 893 items; Scopus, 1873; and Web of Science, 1857. Out of 4623 articles, 4369 were excluded because they were not written in English, they were duplicates, they were not RCT studies and they did not have full text available. Only 19 articles fulfilled inclusion criteria. Furthermore, we excluded six articles after quality assessment according to the Pedro score checklist. Finally, a total of 12 studies were examined for the systematic review (Figure 2). 

The level of evidence of the included studies, according to the Oxford Center for Evidence-Based Medicine (OCEBM), is II [23].

### 3.2. Quality Assessment

Study quality was assessed through the PEDro Scale (Physiotherapy Evidence Database Scale) checklist. Any discrepancies between reviewers were resolved through discussion with the third author. For this review, we selected studies with a ≥6 PEDro score. If the studies’ score was not reported in the PEDro database, two authors assessed the score independently. The researchers were blinded to each other’s quality assessment, and in the event of disagreement, a third opinion was sought. The results of methodological quality assessment are reported in Table 1. 

### 3.3. Synthesis of the Results

A total of 350 patients were involved (mean age 58.36 years), 234 men and 115 women. A summary of the included studies is presented in Table 2. Seven studies focused on upper limb [67,69,70,71,74,76,77], and five on lower limb [68,72,73,75,78].

All the 12 studies included were RCTs. 

Seven studies involved patients recruited from a hospital, a medical center or a rehabilitation clinic [67,68,69,70,71,72,78]; in one study, patients were enrolled from hospital stroke units and local stroke support groups [77]; and in four studies [73,74,75,76], the site of the recruitment was not clearly specified. 

Ten trials included patients with chronic stroke (onset ≥6 months) [69,70,71,72,73,74,75,76,77,78]; one, with post-acute stroke (a stroke within 3 months) [67]; and one, between 2 weeks and 6 months post-stroke [68].

One study focused only on ischemic stroke [71]; eight involved either ischemic or hemorrhagic stroke [67,68,69,72,73,74,74,78]; and three studies did not clarify the stroke etiology [70,75,77]. All the studies revealed the stroke affected side (left/right) except for two studies [75,77].

Six studies stated the first/single nature of the stroke [67,70,72,73,74,76]; one [68], the possible presence of previous stroke; and five studies [69,71,75,77,78] did not specify.

Subramanian et al. used patients that had already participated in the previously trial, [74,76] but evaluated different outcomes. 

### 3.4. Intervention Protocol for Experimental Group

There was a broad range of variability in rehabilitation approach in terms of training duration, frequency and length (Table 2). 

Considering the FIVR tools, the authors used different association of devices. Mekbib et al. [67] used a system called “mirroring neuron VR Rehab” based on a head-mounted display, two HTC Vive tracking stations, Leap motion and a central controller. De RooiJ et al. [68] employed the GRAIL (Gait Real-time Analysis Interactive Lab). Lin et al. [69] employed an Oculus Rift, Leap motion controller and a specific software. Huang et al. [70] used a HTC Vive. Ögun et al. [71] used the leap motion installed on a head-mounted display. Two studies [75,77] were based on a head-mounted display and sensors. Kim et al. [72] and Cho et al. [73] used treadmill training associated with a large screen projection and a VR program. Two studies [74,76] used the CAREN system to recreate the virtual environment and sensors to track the movements and stereoscopic glasses to view the simulated scene on a large projection screen. Kang et al. [78] employed treadmill training associated with a head-mounted display.

Seven trials [70,72,73,75,78] consisted of a two-step rehabilitation protocol. In Mekbib et al. [67], the first phase was a virtual training based on reaching, grasping and releasing a ball in a basket, and this task could be completed either in limb mirroring therapy or affected limb therapy mode. The second phase involved occupational therapy. In Lin et al. [69], patients initially took virtual training based on hand exercises copying movements of a virtual hand and then conventional motor task exercises. In five studies, virtual reality rehabilitation was associated with standard rehabilitation (either conventional physical therapy plus occupational therapy [73] or conventional/physical therapy alone) [70,72,75,78]. In Kim et al. [72], the experimental group was composed by two sub-groups: a virtual training group (in which patients treadmill trained in different virtual environment) and a community ambulation group. In Kang et al. [78], the treadmill training was further separated either in treadmill training with optic flow with head-mounted display or treadmill alone. 

In a study by De Rooij et al. [68], patients trained with Gait Real-time Analysis Interactive Lab (GRAIL) using several virtual environments to stimulate certain functional recovery. 

Ögün et al.’s [71] experimental protocol consisted of four games, each of which targeted at a specific function of the upper limb: grip function, hand and forearm movement to handling items and complete complex gesture. 

Subramanian et al. [74] employed pointing objects in a virtual reality environment using feedback regarding movement speed and trunk movements to understand its effects on motor recovery. Subramanian et al. [76], in a second trial, focused on the influence of the virtual environment on motor recovery.

Crosbie et al. [77] virtual training consisted of various virtual task (“reach to target, reach and grasp, game tasks”) focusing on upper arm functions.

### 3.5. Intervention Protocol for Control Group

One trial [67] was based on occupational training. De Rooij et al. [68] used conventional treadmill training plus functional gait trainings. Lin et al. [69] utilized a standard mirror therapy plus conventional motor training. In Huang et al. [70], the control group underwent an upper limb training plus extra exercises with conventional machines. Ögun et al. [71] used conventional therapy and sham virtual program. Four trials employed conventional and physical therapy [72,75,77,78]. Two trials [74,76] utilized the same protocol of the experimental group but without the virtual environment. Finally, Cho et al. [73] proposed a conventional physical training plus occupational therapy followed by a virtual reality treadmill training for control group.

### 3.6. Side Effects

Side effects were not considered in seven studies [60,62,63,68,69,70,71]. De Rooij et al. [68] reported side effects both in the intervention and the control groups (for example, dizziness, near falls, fatigue, stiffness, pain) but with no interruption of the therapy. Crosbie et al. [77] registered transient dizziness and headache in two patients in the virtual reality group. Lin et al. and Subramanian in both studies reported no side effects in the groups [69,74,76].

### 3.7. Outcome Measure

Upper limb function was assessed through the Action Research Arm Test (ARAT), the Functional Independence Measure (FIM), the Fugl-Meyer Upper Extremity Scale (FM-UE), the Performance Assessment of Self-Care Skills (PASS), the Reaching Performance Scale for Stroke (RPSS), the Wolf Motor Function Test—Functional Assessment Scale (WMFT-FAS), the Motor Activity Log Amount Scale (MAL-AS), the upper limb Motricity Index and the kinematics data for motor performance and movement pattern. The lower limb function was determined by devices that quantify gait kinematic parameters, by a 6 min walking test (6-MWT) and by a 10-meter walking test (10MWT). The balance was evaluated through the Mini-Balance Evaluation Systems Test (Mini-BESTest), the Timed Up and Go test (TUG), the Activities-Specific Balance Confidence (ABC) scale and the Berg Balance Scale (BBS). To assess ADLs, we used the Barthel Index (BI). A neuropsychological assessment was conducted through a questionnaire (motivation task evaluation) and tests (Stroop test, Rey Auditory Verbal Learning Test (RAVLT), Tower of London (TOL), Rey Osterrith Complex Figure (ROCF) copy). De Rooij et al. [68] assessed participation and satisfaction through the restriction’s sub-scale of the Utrecht Scale for Evaluation of Rehabilitation-Participation (USER-P) and through questionnaires regarding physical functioning (Stroke Impact Scale-16), fatigue (Fatigue Severity Scale), anxiety and depression (Hospital Anxiety and Depression Scale), falls efficacy (Falls Efficacy Scale International) and quality of life (Stroke Specific Quality of Life Scale).

**Table 2:** Main characteristics of the included studies.

Author	Patient	Tools	Inclusion Criteria	Training	Intervention	Control Group	Assessment	Outcome
*Upper limb*								
Mekbib et al., 2021 [67]	N = 23 EG 12; CG 11	Mirroring neuron VR Rehabilitation (MNVR-Rehab): HMD, two HTC Vive tracking stations, Leap Motion and ALIENWARE laptop	(1) first ischemic or hemorrhagic stroke with moderate to severe upper limb dysfunction (2) stroke within 3 months (3) age > 18 years (4) neither hearing nor vision deficits (5) MMSE > 16	2 h per day, 4 days a week for 2 weeks	60 min of virtual training (reach, grasp and release colored ball into a basket through MNVR-Rehab) plus 60 min of occupational training	Occupational therapy based on daily living activities, balance control, gait training, weight shift and upper limb functional training	Assessment at baseline and post-intervention (2 weeks). FM-UE; BI	MNVR-Rehab is an encouraging rehabilitation apparatus that may increase upper limb function in subacute stroke subjects compared to occupational therapy
Lin et al., 2021 [69]	N = 18 EG 9; CG 9	Virtual reality mirror therapy (Oculus Rift and Leap Motion Controller and dedicated Software)	(1) 6 months post-unilateral infarction or hemorrhage stroke (2) FMUE between 23 and 60 (3) MMSE >24	Sessions of 50 min, two days per week for 9 weeks	30 min of virtual reality mirror therapy plus 20 min of traditional motor task specific exercises	30 min of conventional mirror therapy plus 20 min of traditional motor task specific exercises	Assessment at baseline and post-intervention (9 weeks). FM-UE assessment	Adding virtual reality to mirror therapy can increase upper limb function in chronic stroke subjects.
Huang et al., 2020 [70]	N = 18 EG 20; CG 20	HTC Vive	(1) first unilateral stroke (after 3 and 24 months) (2) hemiparesis with upper limb dysfunction after stroke (3) upper limb rehabilitation to convalescents levels of Brunnstrom stages III to V (4) able to sit and stand without help ford 2 min (BBS ≥ 3)	20 sessions of 30 min, 3 times a week, over 8 weeks plus 1 h of upper limb conventional training	Upper limb conventional training plus immersive virtual reality gaming (shoot balloon, electric current stick and shooting game)	Upper limb conventional training plus physical training with climbing bar, ball bearing and pulley	Assessment at baseline and post-intervention (8 weeks). FM-UE; BBT; FIM self-care score	In stroke rehabilitation the use of an immersive virtual reality system improves upper limb function.
Ögün et al., 2019 [71]	N = 65 EG 33; CG 32	HMD + Leap Motion	(1) MMSE score ≥ 25 (2) stroke between six and 24 months (3) Modified Ashworth Scale score < 3 (4) upper extremity and hand Brunnstrom score ≥ 4	Sessions of 60 min of therapy, three days per week, for six weeks	immersive VR plus Leap motion training consists of four games (play task-oriented games that aimed at gripping and handling of objects with arm and at forearm motion and stability	45 min of conventional upper extremity active exercises including the same used in the VR group plus 15 min of sham VR training	Assessment at baseline and post-intervention (six weeks). FM-UE; ARAT; FIM, PASS	Applying immersive VR with leap motion in stroke patients has a statistical significance on upper extremity function and daily life activities but not on independence.
Subramanian et al., 2015 [74]	N = 24 EG 12; CG 12	Stereoscopic glasses + projector + screen + 3D virtual environment (CAREN)	(1) between 40 and 80 years (2) single ischemic or hemorrhagic stroke 6 to 60 months previously (3) scored 3 to 6/7 on the Chedoke-McMaster Stroke Assessment arm sub-scale (4) no other neurologic or neuromuscular/orthopedic problems affecting the upper limb and trunk	12 sessions: 3 times per week over 4 weeks	A 3D virtual environment (CAREN system) simulated a supermarket scene. Subjects had to point 6 objects placed just beyond arm’s length, without physically touching them.	Participants had to point at targets in a physical environment	Arm and trunk kinematic assessment at baseline, after 4 weeks, and 3 months following intervention. Neuropsychological assessment only at baseline Neurocognitive assessment (Stroop test, RAVLT, TOL, ROCF copy)	An increase in kinematic data for upper limb motor recovery was related to milder neurocognitive deficits.
Subramanian et al., 2013 [76]	N = 32 EG 16; CG 16	Stereoscopic glasses + projector + screen + 3D virtual environment (CAREN)	(1) between 40 and 80 years (2) single stroke 6 to 60 months previously (3) scored 3 to 6/7 on the Chedoke-McMaster Stroke Assessment arm sub-scale (4) no other neurologic or neuromuscular/orthopedic problems affecting the upper limb and trunk	12 sessions of 45 min over 4 weeks	A 3D virtual environment (CAREN system) simulated a supermarket scene. Subjects had to point to 6 objects placed just beyond arm’s length, without physically touching them.	Participants had to point at targets in a physical environment	Assessment at baseline, after 4 weeks, and 3 months following intervention. FMA, RPSS, WMFT-FAS, MAL-AS mean scores, Motivation Task Evaluation Questionnaire, and kinematic analysis	Both groups improved arm motor impairment measures, clinical impairment scores and activity levels. Improvements can be attributed to practice intensity. VE training led to better results in arm motor recovery, especially in the moderate-to-severe group.
Crosbie et al., 2012 [77]	N = 18 EG 9; CG 9	HMD + desktop computer + motion tracking system + sensors	(1) medically stable (2) 18–85 years (3) 6–24 months following a first stroke (4) able to follow a two-step command	Nine sessions of 30–45 min over three weeks	Virtual reality through an HMD for upper limb training. The virtual tasks simulated a range of upper limb tasks related to reach to target, reach and grasp and game tasks.	Conventional therapy based on muscle facilitation, stretching exercises, strengthening activities and functional tasks	Assessment at baseline, post-intervention, after three weeks, and 6 weeks following the intervention. ARAT and an exit questionnaire.	This pilot study has demonstrated the feasibility of a RCT in chronic stroke patients, if careful consideration is given to the recruitment methods and outcome measures. Larger trials are needed to offer high-quality evidence for the specific effect of virtual reality-mediated therapy in upper limb stroke rehabilitation.
*Lower limb*								
De Rooij et al., 2021 [68]	N = 52 EG 28; CG 24	GRAIL	(1) WHO diagnosis of stroke (2) between 2 weeks and 6 months post-stroke (3) walking without help for balance and coordination (FAC > 3) (4) walking in daily life feeling self-limitation (5) community living (6) between 18 and 80 years	Sessions of 30 min, 2 times a week for 6 weeks	Training on GRAIL several virtual environment with different purpose and variable degree of complexity	10–15 min of treadmill training and 15 min of functional gait training	Assessment at baseline, 6 weeks, and 3 months post-intervention. USER-P; TUG test; 6MWT Walking activity (total number of steps a day, duration of walking activity per day and step frequency); Mini-BESTest; FES-I; SIS-16; FSS, HADS anxiety and depression; SS-QOL	The effect of VRT was not statistically different from the effect of non-VRT on analyzed outcomes in community-living people after stroke, but virtual treadmill training was safe and well-tolerated by patients and therefore could be a useful supplement to stroke rehabilitation
Kim et al., 2015 [72]	N = 27 VRCA-G 10; CA-G 11; CG 7	Treadmill + projector + screen + VR program	(1) 6 months post-stroke with hemiplegia (2) gait speed < 0.8 m/s (3) autonomous walking without device for more than 6 min (4) MMSE-K >24	Sessions of 30 min, 3 times a week for 4 weeks (VRCA-G and CA-G) plus sessions of 30 min, 2 times a day for 4 weeks (all groups)	VRCA G trained with a treadmill in 4 different VR environments (sidewalk walking, overground walking, uphill walking and stepping over obstacles) and a progressive speed increase based on the patient’s condition. CA-G trained in the real world in 4 environments: overground walking, stair walking, slope walking and unstable surface walking. Both groups received also general physical therapy.	General physical training	Assessment at baseline and 4 weeks post-intervention. TUG test; ABC; 6MWT; GAITrite walking	In post-stroke subjects, VR treadmill training-based community ambulation and community ambulation training help enhancing dynamic balance ability, activities-specific balance confidence and temporal/spatial gait or gait endurance. These methods are both useful to increase functional skills but, due to VR effects on patient’s physical and psychological fields, it would be more beneficial using this therapy before the community ambulation training.
Cho et al., 2015 [73]	N = 22 EG 11; CG 11	Treadmill + projector + screen + VR program	(1) single stroke, (2) 6 months post-stroke, (3) able to walk 10 m with and without the use of an assistive device, (4) able to understand and follow simple verbal instructions (Korean version of the MMSE score > 24) (5) no severe heart disease or uncontrolled hypertension.	Sessions of 30 min a day, 5 times a week for 4 weeks	VR training (treadmill training) with four cognitive load tasks (memory, arithmetic and two verbal tasks)	VR training (treadmill training)	Assessment at baseline, 3 days after the last experimental training. GAITRite walkway system for spatiotemporal gait parameters under single and dual task conditions.	Beneficial effect of VRTCL on walking function under single and dual task conditions in chronic stroke patients. In the VRTCL group there was a greater increase in walking function during the dual task condition than in the control group.
Lee et al., 2014 [75]	N = 21 EG 10; CG 11	HMD	(1) chronic stroke (2) no medications influencing balance (3) MMSE score < 24 (4) no pain or disability associated with acute musculoskeletal diseases (5) able to sit for over 10 s without help (6) able to stand without help for 1 min	20 sessions of 30 min over 4 weeks for all participants + 20 sessions of 30 min over 4 weeks for EG	16 exercises to train postural control organized on three degrees of difficulty (lying, sitting and standing) plus standard rehabilitation	Standard rehabilitation	Assessment at baseline and after 4 weeks post-intervention. TUG test; BBS; GAITrite walkway	Adding VR training to conventional rehabilitation leads an increase in a number of gait parameters (gait velocity, step length and stride length) compared with traditional therapy alone.
Kang et al. [78]	N = 30 TOF: 10; Treadmill G: 10; CG: 10	HMD + treadmill	(1) hemiparetic stroke 6 months after diagnosis, (2) able to walk without help for more than 15 min, (3) no visual deficits or hemianopsia, (4) MMSE ≥ 21 (5) Brunnstrum stage > 4	Sessions of 30 min, 3 times a week for 4 weeks plus conventional therapy 5 times a week for four weeks	Treadmill training with an HMD showing walking on a street with a progressive increase in speed plus conventional therapy. Treadmill group experienced treadmill without extra device and with a progressive rise of speed plus conventional therapy.	Stretching added range of motion training plus conventional therapy	Assessment at baseline and 4 weeks post-intervention. TUG test; FRT; 10 MWT; 6MWT	Treadmill training with optic flow can help improve balance and gait function in chronic post-stroke patients especially taking advantage of optic flow speed modulation.

### 3.8. Upper Limb Function

Mekbib et al. [67] presented a significant between groups difference in terms of FM-UE (*p* = 0.007) after virtual reality treatment, whereas there was no statistical difference in terms of BI (*p* = 0.193). Lin et al. [69] demonstrated an improvement in the VR mirror therapy group regarding total score and hand component of FM-UE (*p* = 0.033 and *p* = 0.008, respectively) compared to mirror therapy group. Huang et al. [70] demonstrated a significant difference in FM-UE between virtual reality therapy and conventional therapy (*p* = 0.014 vs. *p* = 0.021). Ögün et al. [71] examined as the primary outcome the FM-UE, while, as the secondary outcome, the ARAT, and showed an improvement in both upper limb measures, which was higher in the virtual reality group compared with the control group (*p* < 0.001). Moreover, FIM and PASS results were statistically different, with better results in the virtual reality group than in the control group (*p* < 0.001). However, matching these scores with the minimal clinically important difference (MCID), the authors proved a no statistical difference regarding FIM values, whereas PASS scores were statistically significant since there was no MCID PASS score limit. Subramanian et al. [74] analyzed kinematic data from arm and trunk movements, and the study showed that the virtual reality group had better results than the control group in terms of motor functioning (*p* < 0.05) and smoothness of movements (*p* < 0.001) (the experimental group showed a lower degree of counteracting trunk movements during pointing). Furthermore, the authors demonstrated that these results were also correlated with minor cognitive deficits in memory, attention, visual perception capacity and problem solving.

Subramanian et al. [76] analyzed the RPSS, the WMFT-FAS and the MAL-AS as clinical outcomes and also assessed kinematic outcomes through motor performance (end point velocity and precision) and movement pattern (joint angular excursions, trunk displacement) in two sub-groups of subjects (mild and moderate–severe motor impairment according to the FMA), both in the virtual reality group and the control group. Experimental and control groups had better outcomes in end point velocity (*p* < 0.05) and shoulder horizontal adduction for the lower–middle target (*p* < 0.01), in the RPSS elbow sub-scale (close target: *p* < 0.02; far target: *p* < 0.05) and in the WMFT-FAS (*p* < 0.05). Virtual group showed superior “target-specific changes” [76] during shoulder horizontal adduction, ensuing correct targeting for lower–middle and upper–ipsilateral targets (*p* < 0.01). Only the moderate to severe virtual reality group showed an increase in MAL-AS (*p* < 0.05). Crosbie et al. [77] investigated the Upper Limb Motricity Index and the ARAT. The authors found no clinically differences in the groups for both the measures (*p* = 0.485 and *p* = 0.139) due, probably, to a low sensibility of the evaluation: in top-performing patients, these measures cannot distinguish minor and moderate variation. 

### 3.9. Lower Limb Function

De Rooij et al. [68] assessed as the primary outcome the influence of virtual reality treadmill training on participation through the USER-P. Focusing on balance and gait performance, the authors considered TUG test, 6-MWT and Mini-BESTest. They showed a statistical difference regarding participation (*p* < 0.001) and dynamic balance (Mini-BESTest *p* < 0.001) at T1, but no statistical between group difference in participation (*p* = 0.221), TUG test (*p* = 0.453), 6-MWT (*p* = 0.144) and Mini-BESTest (*p* = 0.721).

Kim et al. [72] assessed balance through the TUG test for dynamic balance and ABC and gait through the 6-MWT test and GAITRite (CIR System Inc., Franklin, NJ, USA, 2008) in virtual reality, community ambulation and control group. A statistical improvement was found on TUG, ABC and 6-MWT, both in the virtual reality group and the community ambulation group (VR: TUG *p* = 0.001, ABC *p* = 0.018, 6-MWT *p* = 0.007; CA: TUG *p* = 0.000, ABC *p* = 0.000, 6-MWT *p* = 0.004), while no significant difference was found in the control group. Regarding temporal and spatial gait data between groups difference, there was a statistical difference on TUG (*p* = 0.048) and ABC (*p* = 0.043) between the virtual reality and control groups.

Cho et al. [73] studied the lower limb function through spatio-temporal gait values. They showed that using virtual reality, integrated or not with cognitive load, has positive influence on locomotor function under single task situation with an improvement in gait speed (*p* = 0.000), cadence (*p* = 0.000), step length (*p* = 0.000) and stride length (*p* = 0.000). During dual task situation, all the parameters of locomotion improved in both groups (*p* < 0.05), with higher results in the group with the integration of virtual reality training and cognitive load (*p* < 0.05). Lee et al. [75] evaluated balance trough TUG and BBS and gait performance through GAITRite. The study proved a significant difference in virtual reality group on TUG (*p* = 0.011), BBS (*p* = 0.007) and gait parameters such as velocity (*p* = 0.013), cadence (*p* = 0.047), step length and stride length of paretic (*p* = 0.009 and *p* = 0.010) and non-paretic sides (*p* = 0.007 and *p* =0.006). In the control group, there was a statistical improvement in TUG (*p* = 0.038), step length (*p* = 0.037) and stride length (*p* = 0.022) on the paretic side and stride length on the non-paretic side (*p* = 0.049). Considering the group x time exchange, a significant improvement was found in gait velocity (*p* = 0.030) and step length and stride length on both the paretic (*p* = 0.042 and *p* = 0.029) and non-paretic sides (*p* = 0.011 and *p* = 0.018). Kang et al. [78] used TUG, FRT, 10MWT and 6MWT as outcome measures. TUG values were better in the treadmill training with the optic flow group compared with standard treadmill and control group (*p* < 0.05). There was a statistical difference in the FRT between the treadmill training with optic flow and control groups (*p* < 0.05). Regarding gait performance, there was a significate variation in terms of 10MWT and 6MWT between the optic flow group and the other two groups (*p* < 0.05).

## 4. Discussion

Immersive virtual reality treatment has great potential in motor stroke rehabilitation and can offer additional benefits in comparison with standard therapy, both on upper and lower limbs. Upper limb deficits are a common consequence of stroke: over 80% of stroke survivors have an upper limb dysfunction, e.g., spasticity, dystonia, muscle contracture, loss of strength and dexterity, decreased active joint range of motion, lack of precision and bi-manual coordination [79]. Furthermore, walking deficits are also frequently observed in stroke survivors, and a structured rehabilitation plan is shown to enhance the independence in locomotor function in about 50% of the patients. In contrast, in more than 70% of patients persist some level of gait deficits [80]. Moreover, patients complain about a reduction of muscle strength and a failure of intentional actions that further affect locomotion and participation [81]. Therefore, walking rehabilitation is one of the major goals to achieve during the following 12 months after stroke [80]. In addition, the risk of fall is high, and approximately 70% of stroke patients experience a fall in the sub-acute phase after a stroke. This is probably due to a compromised balance, mainly vertical equilibrium, that causes “temporal inter-limb asymmetries” in 48–82% of patients and spatial anomalies in 44–62% of patients [81].

Previous studies already assessed the effectiveness of virtual reality in stroke patients [82,83,84]. In particular, a recent review by Patsaki et al. [84] investigated the effectiveness FIVR in stroke patients limited to HMD. The aim of the present review was to include all forms of FIVR, considering only high-quality RCTs.

The results of the present review show that FIVR has a beneficial effect on gait performance and dynamic balance of lower limbs [72,75]. Although De Rooij et al. [68] found no statistical difference in TUG, 6MWT and Mini-BESTest between the virtual group and the control group, they concluded that virtual reality could be benefit in stroke rehabilitation, because it was positively rated by patients, well-tolerated and applicable in clinical practice, with limited adverse events, and had the possibility to customize the intervention based on the levels of adherence.

Furthermore, the studies considered in the present review demonstrated interesting results of FIVR on functioning, control and proprioception of the upper limb in post-stroke patients using both the clinical scale and instrumental analysis. In particular, there was a statistical significant improvement in FM-UE in all the studies that analyzed this outcome [62,63,66], and in kinematic parameters [74,76], which represents an objective and detailed evaluation of articular range of motion [85].

Moreover, evidence supports the synergic integration of FIVR and standard care rehabilitation. In details, Mekbib et al. [67] developed a system in which virtual reality was associated with a mirror therapy during occupational therapy and showed higher results on upper limb recovery compared with occupational therapy alone. Lin et al. [69] proposed a virtual reality mirror therapy with a significant difference in upper limb function improvement compared with mirror therapy alone. In addition, FIVR has been applied with treadmill for gait training, and although there was equivalence on functional outcomes, FIVR represents a valuable addition to stroke rehabilitation as a safe and well-tolerated therapy that improved the compliance of the patients [68,72]. Kim et al. proposed FIVR as an intermediate phase before community de-ambulation to improve spatial gait and gait endurance. The positive effect on gait parameters (gait velocity, step length and stride length) was also confirmed by Lee et al. Moreover, in FIVR, it is possible for the addition of a cognitive load task to reproduce the environment and situations similar to real community activities [73]. Furthermore, by integrating visual feedback on FIVR gait training, the patients are able to self-correct gait deficits, by analyzing the postural and balance control suggestions showed in the virtual environment [75,86]. FIVR is defined by authors as a low-cost, portable tool that could be integrated in the rehabilitation plan of post-acute and chronic patients. However, the improvement of movement detection systems and the development of personalized software that includes exercises built on the patient’s needs still represent a challenge [84]. In this context, the collaboration between the medical and entertainment industries could provide interesting solutions on the development of virtual protocols of exercise that are even more realistic and inclusive [70].

One of the advantages of FIVR is the potential use to support and integrate neurocognitive approaches in neurorehabilitation, such as action recognition, motor imagery and mirror therapy [87,88,89,90]. All these approaches share the neurophysiological evidence that the motor system goes beyond the idea that is a mere implementer of motor action, but it plays a role in cognitive functions such as action recognition, motor imagery (MI) and processing the action content of words. As a whole, the motor system is recruited whenever actions are executed, imagined, recognized or verbally described [91,92,93,94]. This capacity to use the neural structures involved in action execution also for cognitive strategies as action recognition and MI is also defined as action re-enactment [95]. Within this theoretical framework, new methods have been proposed, known as cognitive-based strategies or mental simulation practices (MSP), which have the potential to target the central representation of the gait pattern. MI and AO are the most common MSP [11]. In this context, FIVR could improve MSP, with a more immersive experience, favoring the restorative approaches that re-enact the motor representations (top–down approach) rather than approaches focusing on peripheral components (bottom–up approach), due the stimulation of mirror neurons that enhance the reorganization of the damaged cortex, decrease in cortical hyperexcitability and synthesis of neurotrophic factors, boosting dendritic spine formation and axonal sprouting [96]. According to Arcuri et al., it is possible to integrate non-invasive and portable neuroimaging methods, such as EEG to monitor patients during FIVR for a more targeted rehabilitation approach [97].

Furthermore, as highlighted in previous studies, FIVR showed positive medium-term effects in improving post-stroke depressive symptoms, increasing motivation, augmenting the variability of neurorehabilitation. When patients are motivated, they actively participate in rehabilitation and are more concentrated on completing the task requested [84,98]. Moreover, the integration of tactile sensations and peripheral nerve activation could favor the rearrangement of the cortical connections and, consequently, improve motor functionality [99].

Although significant between groups, differences are not demonstrated in all the studies considered, and the results of the present review, in line with a previous review of Patsaki et al., support the benefits of FIVR in rehabilitation of post-stroke patients [84]. In detail, FIVR has been shown to have positive effects on upper and lower limb function when compared to or integrated with standard rehabilitation.

In the near future, it is expected that the number of articles on FIVR will increase significantly, due to the rapid technologic development, providing new tools, with possible application in home-based settings for a telemedicine monitoring and training. Moreover, artificial intelligence could be exploited to deliver the right dose of home exercise at the right level and to develop easy-to-use devices that the patient or the care-giver can manage at home [100].

This systematic review has some limitations. First, the studies employed different devices and a small sample size of patients. A larger number of subjects improves the generalizability of the results. Second, there were no longitudinal studies with the examination of long-term effects of FIVR. Moreover, the high amount of withdrawal due to patients’ compliance and recruitment problems, and the single center design of the studies limit the external validity. In the future, multi-center studies could be helpful to better delineate the efficacy of FIVR in stroke survivors.

## 5. Conclusions

Considering the effects of stroke on cerebral tissue and the correlated motor impairment, the employment of virtual reality rehabilitation is founded on the assumption that replication of known tasks through repetitive functional exercise can assist and enhance neuroplastic activation. The results of the present review support the use of FIVR in the rehabilitation plan of stroke patients, constituting an adaptable, multi-faceted rehabilitation method that can target patients’ capacity and elicit positive responses. Moreover, due to the deep engagement of the patient in a virtual environment FIVR could be considered as a complementary tool to improve the effects of cognitive approaches in neurorehabilitation.

Although the potential of FIVR has still to be fully explored, it represents an innovative and engaging rehabilitation tool with beneficial effect for motor recovery, function and quality of life in stroke survivors.

## Figures and Tables

**Table 1:** Studies quality assessment (PEDro Scale).

	Eligibility Criteria	Random Allocation	Concealed Allocation	Baseline Comparability	Subjects Blinding	Therapist Blinding	Assessor Blinding	Adequate Follow-Up (>85%)	Intention-to-Treat Analysis	Between-Group Comparisons	Points Estimates and Measure of Variability Provided	Total PEDro Score	Sample Size ≥ 50
Mekbib et al. [67]	x	x	x	x			x			x	x	6/10	no
De Rooij et al. [68]	x	x	x	x			x	x	x	x	x	8/10	no
Lin et al. [69]	x	x	x	x			x	x	x	x		7/10	no
Huang et al. [70]	x	x		x			x	x	x	x	x	7/10	no
Ögün et al. [71]	x	x	x	x	x		x	x		x	x	8/10	no
Kim et al. [72]		x	x	x			x	x		x	x	7/10	no
Cho et al. [73]		x	x	x			x	x		x	x	7/10	no
Subramanian et al. [74]		x	x	x			x	x		x	x	7/10	no
Lee et al. [75]		x		x			x	x	x	x	x	7/10	no
Subramanian et al. [76]		x	x	x			x	x		x	x	7/10	no
Crosbie et al. [77]	x	x	x	x			x	x	x	x	x	8/10	no
Kang et al. [78]	x	x	x	x			x	x		x	x	7/10	no

## Data Availability

Not applicable.
